# The effects of a brand-specific, hemp-derived cannabidiol product on physiological, biochemical, and psychometric outcomes in healthy adults: a double-blind, randomized clinical trial

**DOI:** 10.1080/15502783.2024.2370430

**Published:** 2024-06-21

**Authors:** Gianna F. Mastrofini, Bridget A. McFadden, Alexa J. Chandler, Blaine S. Lints, Harry P. Cintineo, Nathaniel D. Rhoades, Caroline S. Vincenty, Sten O. Stray-Gundersen, Abbi D. Lane, Shawn M. Arent

**Affiliations:** a University of South Carolina, Department of Exercise Science, Columbia, SC, USA; b City University of New York, Department of Family, Nutrition, and Exercise Science, Queens College, Flushing, NY, USA; c Lindenwood University, Department of Kinesiology, Saint Charles, MO, USA; d University of Michigan, School of Kinesiology, Ann Arbor, MI, USA

**Keywords:** Cannabis, cytokines, pain, profile of mood states, sleep, perceived stress

## Abstract

**Background:**

Cannabidiol (CBD) is a non-psychoactive phyto-cannabinoid derived from the *Cannabis sativa* plant. CBD exhibits various interactions at receptor sites, prompting the research of its potential anti-inflammatory, immunomodulatory, psychological, and pain-relieving effects. This study aimed to investigate the physiological, biochemical, and psychometric effects of a brand-specific, hemp-derived CBD product in healthy adults over a 12-week observation period.

**Methods:**

54 healthy males and females (age = 25 ± 7y; BMI = 24.82 ± 3.25 kg/m^2^) recruited from a large Southeastern University completed the study. Participants arrived at the laboratory after > 8 h of fasting, and > 48 h without alcohol consumption and vigorous exercise. Following baseline measurements (height, weight, blood pressure, electrocardiogram (ECG), and blood work), participants were stratified by sex and randomized to either CBD or placebo groups. Products were administered double-blinded, with both given in liquid form containing medium-chain triglyceride oil, while the CBD product specifically contained 50 mg/mL of CBD. Participants were instructed to consume 1 mL of their product twice daily and were given enough product to last until their next laboratory visit. Data were collected at baseline and on days 30 ± 3, 60 ± 3, and 90 ± 3. Blood was drawn for analysis of immune and inflammatory biomarkers. Chronic pain among participants was calculated using urine samples according to the foundational pain index (FPI). Self-reported psychometric questionnaires were utilized (Cohen’s Perceived Stress Scale, Pittsburgh Sleep Quality Index, Profile of Mood States,10-item Likert scale for perceived pain) to assess stress, sleep quality, mood state, and body discomfort. To determine overall wellbeing, participants completed a daily survey indicating if they missed work or school due to illness. Change from baseline was calculated for each measure, and mixed effects models were used to determine differences between groups over time while adjusting for baseline values (α = 0.05). Data are presented as mean ± standard deviation.

**Results:**

There were no Group-by-Time interactions or Group or Time main effects for immune or inflammatory biomarkers (*p* > 0.05). Analyses revealed no Group-by-Time interactions or main effects observed for perceived stress, sleep quality, overall mood disturbance, and all the profile of mood state subscales (*p* > 0.05), except “vigor-activity.” A Time main effect was found for the sub-score for “vigor-activity” (*p* = 0.007; Pre CBD = 19.5 ± 5.2, Post CBD = 17.3 ± 5.3; Pre PL = 19.0 ± 5.7, Post PL = 17.9 ± 7.1), which decreased from Visit 3 to Visit 4 (*p* = 0.025) and from Visit 3 to Visit 5 (*p* = 0.014). There was a Group main effect for FPI (*p* = 0.028; Pre CBD = 11.9 ± 14.4, Post CBD = 8.8 ± 10.9; Pre PL = 9.0 ± 14.2, Post PL = 12.9 ± 11.5), indicating that the placebo group had greater increases in pain over the intervention compared to the CBD group. No significant differences were found between groups in the incidence and prevalence of “colds or flus” (*p* > 0.05).

**Discussion:**

CBD was safe and well tolerated in healthy adults. These findings show pain was lower in the CBD group, suggesting a potentially positive effect for consumption of CBD. “Vigor-activity” decreased across the intervention, which may be a confounding effect of the academic semester. While the dosage chosen was safe, more research may be warranted using higher doses as these may be needed to observe further therapeutic effects in healthy populations.

## 1. Background

The Cannabis sativa plant contains several phyto-cannabinoids, of which Δ9-tetrahydrocannabinol (THC) and cannabidiol (CBD) are the most widely known. THC exerts its effects on cannabinoid (CB) receptors and is characterized by the psychoactive components typically associated with cannabis. CBD lacks psychotropic activity and has a low affinity for CB-1 and CB-2 receptors [1]. CBD also appears well-tolerated in humans when administered orally up to 1500 mg/day [2]. Recently, hemp, a specific variety of *Cannabis sativa*, and its derivatives (cannabis with ≤ 0.3% THC), were reclassified and are no longer controlled substances under the Farm Bill of 2018 in the United States [3]. Despite limited evidence in humans, CBD is currently purported to have a myriad of physiological and psychological benefits. Considering its affinity for several other neuromodulating target sites outside of CB receptors, CBD may have several health-related applications. However, more rigorous scientific investigation is needed to elucidate these potential effects in humans.

### 1.1. Inflammation/angiotensin converting enzyme/immune system

CBD’s diverse range of interactions at different receptor sites potentially induces a wide range of therapeutic effects on immune function, chronic disease, pain, and inflammation. The anti-inflammatory and immunomodulatory effects of CBD have been researched in situations where tumor necrosis factor (TNF)-alpha, interleukin (IL)-1, and interferon (IFN)-gamma expression were modified and chemokine production was halted by CBD in human cell lines [4,5]. A systematic review found 23 out of 24 preclinical investigations reported reductions in at least one inflammatory cytokine, suggesting CBD contributes to anti-inflammatory effects in many diseased states [6]. CBD may benefit immune function and has been investigated for preventing and treating viral diseases [7] using in vitro models. Wang et al.. (2020) hypothesized, based on its potential to positively influence inflammation and gene expression, high-CBD *Cannabis sativa* extracts could decrease the expression of angiotensin converting enzyme (ACE)-2. The investigators identified 13 high-CBD *Cannabis sativa* extracts that decreased ACE2 protein levels [8].

CBD’s potential analgesic and pain-relieving properties have also garnered interest [9,10]. However, most of these studies used medical cannabis containing a higher THC content with less CBD [11], leading to limited conclusions regarding CBD’s isolated analgesic effects. Investigations considering the use of CBD to improve neuropathic pain has generated conflicting results [12,13]. The generalizability of these studies is limited due to a wide range of cannabinoids and/or cannabis in individuals with preexisting comorbidities.

### 1.2. Psychological measures

CBD may possess anxiolytic and antidepressant properties [2,14–16]. Its influence on serotonin type 3 (5-HT3) receptors has been investigated for potential use as an anxiolytic [17–20]. CBD is widely utilized for sleep support, yet there is limited evidence of its efficacy in improving sleep quality. Acute CBD administration (40 mg/kg) increased sleep duration at night and alertness during waking hours in rodents [21,22]. Further, there appears to be a dose-response effect of CBD supplementation on sleep, with higher doses inducing a sedative effect and lower doses promoting alertness [23]. In a study investigating the impact of CBD on sleep among adults with sleep disturbances, more than half of the 1,793 participants improved sleep quality following the administration of 15 mg CBD over five weeks [24]. Several studies have demonstrated the sedative effects of CBD in clinical populations, however, more evidence is needed in healthy populations.

Research demonstrates positive therapeutic effects of CBD in a wide range of applications; however, there is still a dearth of scientific evidence. The few clinical studies investigating the effects of CBD were limited by small sample sizes, included only clinical populations, and/or failed to investigate prolonged CBD use. However, CBD has gained popularity in a variety of healthy populations, leaving an apparent gap in the literature on CBD’s effects in these individuals. Therefore, further investigation is required to examine the use of CBD for its wide range of purported benefits and investigate potential health implications associated with regular use of CBD. The current study aimed to explore the physiological, biochemical, and psychometric impacts of a brand-specific hemp-derived CBD product in healthy adults over 12 weeks compared to a placebo product. It was hypothesized that ingestion of the CBD product would improve serum ACE expression, mood, and sleep quality, and produce anti-inflammatory, analgesic, anxiolytic, and immunomodulating effects compared to placebo.

## 2. Methods

### 2.1. Experimental approach

**Figure 1.**
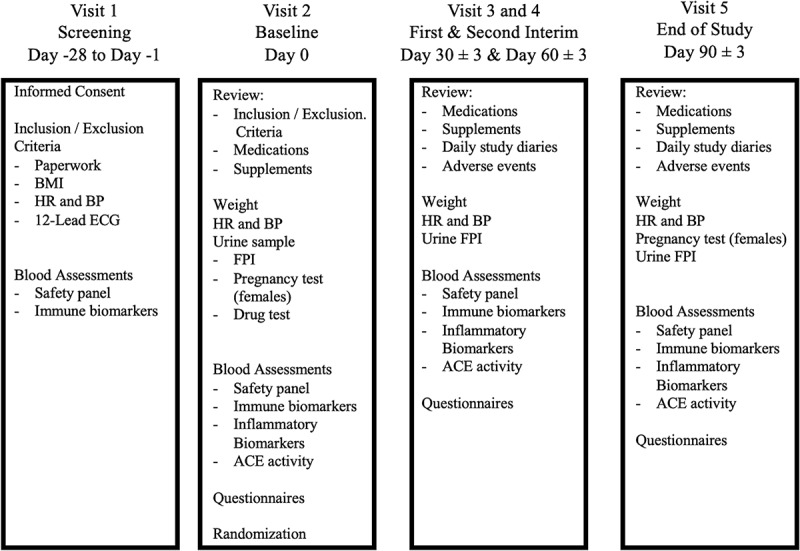
Study design: Visit schedule.

A randomized, double-blind, placebo-controlled study was conducted to assess the impact of CBD on healthy adults. For the primary outcome of mean differences in ACE, based on an alpha level of 0.05, 24 participants per group were needed. Participants attended five visits at the University of South Carolina Sport Science Laboratory (SSL), between January 2022-September 2022 (see Figure 1). Participants arrived after an overnight fast of ≥8 hours and having abstained from alcohol for ≥48 hours prior to all visits. Eligible participants were randomized into one of two groups: test product containing CBD (CBD) or a placebo product (PL). Participants consumed their assigned study products daily and returned to the laboratory on days 30 ± 3, 60 ± 3, and 90 ± 3 for follow-up assessments. This study was approved by the University of South Carolina Institutional Review Board, and informed consent was obtained for all participants (Pro00115662) and was registered at ClinicalTrials.gov (NCT05212402).

Each box represents the schedule of events for each of the 5 study visits.

### 2.2. Study design and supplementation protocol

#### 2.2.1. Visit 1 (screening visit)

After providing written informed consent, eligibility was confirmed by a researcher. Inclusion and exclusion criteria are displayed in Table 1. Participants completed medical history questionnaires and disclosed current medications and dietary supplements. Participants were instructed to continue taking all current medications and supplements for the duration of the study. Height and body weight were measured using a stadiometer and calibrated scale (Health-o-meter Professional, Pelstar LLC, Alsip, IL, USA). A 12-lead electrocardiogram (ECG) was performed with the participant in the supine position, and the absence of likely pathologic cardiac rhythm abnormalities was confirmed by an exercise physiologist PhD with cardiovascular system expertise and extensive training in ECG interpretation. Heart rate and blood pressure were measured following the procedures detailed below. Lastly, a blood sample was obtained to assess safety-related biomarkers listed in Table 2.

**Table 1.** Exclusion criteria.

Exclusion Criteria
Have a known sensitivity or allergy to any of the investigational products or their ingredients.
Female participants who are lactating, pregnant or planning to become pregnant, or male participants of reproductive potential in a heterosexual relationship planning a pregnancy as confirmed at the baseline visit.
Documented medical history of immune disorder, hepatitis B or hepatitis C, or reported immune disorder diagnosis.
Active psychiatric disorder requiring hospitalization within the 12 months prior to screening or currently on medication(s) to treat any psychiatric disorder(s).
Any cognitive impairment that would preclude study participation or compliance with study procedures (e.g. Alzheimer’s, dementia).
History of malignancy or those with any first-degree relatives with a history of cancer (e.g. familial cancer disorders) within 5 years.
History of clinically significant cardiovascular, respiratory, renal, cerebrovascular, metabolic, pulmonary, gastrointestinal, neurological, hematological, autoimmune, lymphatic, psychiatric, chronic pain and sleep disorders, hepatobiliary (with the exception of Gilbert’s syndrome or asymptomatic gallstones) or endocrine disorders, or other clinically significant medical condition that may preclude safe study participation.
Participants with controlled or uncontrolled hypertension including stage 1 hypertension (systolic blood pressure ≥129 mmHg and diastolic blood pressure ≥89 mmHg).
Participants who are on medications as prescribed for any of the aforementioned exclusionary criteria. Participants on stable dose of thyroid medication (no dosage changes within last 3 months) are acceptable.
Consumption of prescription or non-prescription: angiotensin converting enzyme inhibitors, angiotensin receptor blockers, barbiturates, cocaine, ethanol, selective serotonin reuptake inhibitor, protease inhibitors, warfarin, sildenafil, theophylline, tricyclic antidepressants.
Receipt or use of an investigational product in another research study within 30 days or 5 half lives (whichever is longer) prior to baseline (Visit 2) or currently participating in another study.
History of alcohol or substance abuse in the 12 months prior to baseline visit (Visit 2).
Current or recent use (within one month prior to Visit 2) of cannabis (e.g. marijuana) or cannabis related products (e.g. CBD) in any ingestible or inhalable forms.
Positive urine drug test for THC or drugs of abuse (Amphetamine, cocaine, marijuana, methamphetamine, and opiates) at baseline (Visit 2).
Safety blood tests at screening more than 2 times the upper limit of normal (ULN) for liver or kidney function tests.
Evidence of clinically significant anemia (as judged by the Investigator) on screening hematological testing.
Fasting blood glucose of ≥160 mg/dL (after a repeat that confirms the original result) at screening.

**Table 2.** Immune system, safety endpoint, and Foundational Pain Index biomarkers.

Immune System Markers	Comprehensive Metabolic Panel	Foundational Pain Index Urinary Biomarkers
Absolute Monocytes Absolute Eosinophils Absolute Neutrophils Absolute Basophils Absolute Lymphocytes Hemoglobin (HGB) Hematocrit (HCT) Mean Corpuscular Volume (MCV) Mean Corpuscular Hemoglobin (MCH) Mean Corpuscular Hemoglobin Concentration (MCHC) Platelets White Blood Cell Count (WBC) Red Blood Cell Count (RBC) Red Blood Cell Distribution Width (RDW) Mean Platelet Volume (MPV)	Blood Urea Nitrogen (BUN) Albumin Calcium Potassium Alkaline Phosphate (ALK) Aspartate Aminotransferase (AST) BUN/Creatinine Ratio Chloride Creatinine Fasting Blood Glucose Potassium Sodium Total Bilirubin Total Protein	Quinolinate Kyrurenate methylmalonic acid xanthurenate homocysteine 3-HPMA Vanilmandelate 5-HIAA Hydroxymethylglutarate Pyroglutamate Ethylmaolnate

#### 2.2.2. Visit 2 (baseline visit: Day 0)

Eligible participants returned for the baseline visit, and eligibility was reassessed to ensure all inclusion and exclusion criteria were met. Body mass, heart rate, and blood pressure were measured. Urine samples were collected to confirm the absence of illicit drug use and negative pregnancy status (First Response Pregnancy Test, Church & Dwight, Ewing Township, NJ, USA). The remaining urine was sent for Foundational Pain Index (FPI) analysis detailed below. A blood draw was completed to assess safety and efficacy markers (see Table 2). Participants then completed a series of questionnaires detailed below.

After baseline data were collected, participants were stratified by birth sex and randomized to receive either the test product (CBD = 28 [males: *n* = 14; females: *n* = 14]) or placebo (PL = 28 [males: *n* = 14; females: *n* = 14]). The randomization scheme was generated using SAS 9.4 PROC PLAN with the default procedure for random number seed generation. Participants were provided with a high-fat food due to the increased bioavailability of CBD when ingested with fat [25] and consumed their first dose of the study product. Participants were monitored by the research team for adverse reactions to the study product for 15 minutes post-ingestion. Participants were then provided with enough study product to last 45 days and were educated on entering the daily study diary to measure compliance, health, and supplement/medicine usage.

#### 2.2.3. Visit 3 & visit 4 (interim visits: day 30 ± 3 & day 60 ± 3)

Participants returned to the SSL for each interim visit and brought back the remaining study product, which was used to calculate compliance along with direct questioning and daily study diaries. Those who were non-compliant (<70% of the expected volume consumed at Visit 3 and < 80% at Visit 4) were reeducated on proper product administration.

Adverse events and changes in health, supplements, or medications recorded in study diaries were reviewed with the participant. Body mass, heart rate, and blood pressure were measured, and participants provided a urine and blood sample. Participants were provided a high-fat snack and consumed their first daily dose of the newly distributed study product while the research team monitored them for 15 minutes for any adverse reactions. During this time, participants completed the assigned questionnaires. Participants were then provided with enough study product to last 45 days.

#### 2.2.4. Visit 5 (final visit: day 90 ± 3)

Upon arrival, remaining study products and diaries were collected. Adverse events and changes in health, supplements, or medications during the study period were reviewed. Bodyweight and vital signs were recorded. Participants provided blood and urine samples. A portion of the urine sample was used to confirm negative pregnancy status. Lastly, participants completed the study questionnaires.

### 2.3. Study products

Study products were administered orally in liquid form with medium-chain triglyceride oil and natural flavors (terpene in very low quantities). The placebo product matched the test product regarding appearance, taste, and smell. The CBD test product included 50 mg/mL of CBD with THC levels of < 0.3% 3, , and CBD content was verified by an independent laboratory. Participants were instructed to consume 1 mL of the product sublingually twice per day, approximately 12 hours apart, for a daily dosage of 100 mg CBD. Participants were instructed to consume their study product immediately after a high-fat meal (60–75% fat) and refrain from eating or drinking for five minutes after consuming the study product.

### 2.4. Measures

#### 2.4.1. Vital signs

Heart rate and blood pressure were measured using an automated blood pressure cuff (HEM 907XL; Omron Electronics LLC, Hoffman Estates, IL, USA) [26]. Participants rested in an upright seated position for 5 minutes prior to measurements. The investigator placed the cuff around the proximal portion of the right arm in line with the brachial artery. After the first recording, the cuff was removed for one minute, placed again, and blood pressure was re-recorded. If the two values differed by more than 10%, a third value was obtained. The average systolic and diastolic pressures of the two closest values were used along with the corresponding average heart rate.

#### 2.4.2. Blood collection

Approximately 12 mL of blood was collected from the antecubital vein using standardized phlebotomy techniques at each visit. Blood was drawn into plastic serum-specific separator vacutainer tubes (SST; Becton, Dickinson and Company, Franklin Lakes, NJ, USA) and dipotassium ethylenediaminetetraacetic acid tubes (K2 EDTA; Becton, Dickinson and Company, Franklin Lakes, NJ, USA). Tubes were inverted 8 times after blood was collected. SST tubes were allowed to sit for 30 minutes prior to centrifugation at 1600×g for 15 min (642E; Drucker Diagnostics, Port Matilda, PA, USA). Serum was transferred into 1.5 mL aliquot tubes. Approximately 5 mL of serum was stored at −80°C for analysis of inflammatory markers (TNF-α, IL-10, and IL-6) using commercially available magnetic bead assay kits (Human TH17 Multiplex Assay, EMD Millipore Corporation, Burlington, MA, USA) and a magnetic multiplex analyzer (MAGPIX, Luminex, Austin, TX, USA). Average CV% were calculated for the inflammatory markers (TNF- α: 7.4%, IL-10: 6.6%, and IL-6: 6.4%). The remaining ~7 mL of serum and whole blood samples were sent to a CLIA-certified laboratory (Bio-Reference Laboratories, Inc. Elmwood Park, NJ, USA) for analysis of safety endpoint and immune system biomarkers (see Table 2).

#### 2.4.3. Urine collection

Midstream urine samples were collected and sent to Ethos laboratory for analysis. Chronic pain index among participants was calculated using urine samples at Visits 2, 3, 4, and 5 according to the FPI developed by the Ethos laboratory (Newport, Kentucky) [27]. FPI is a score from 0–100 that is derived from measurements of 11 biomarkers (see Table 2) in urine that are associated with biochemical pathways involved in the pathogenesis of chronic pain; higher scores indicate higher chronic pain. The biomarkers are associated with chronic inflammation, nerve health, neurotransmitter status, and oxidative stress. The levels of the biomarkers were measured and tabulated using a proprietary algorithm to generate the FPI score.

#### 2.4.4. Questionnaires

Cohen’s Perceived Stress Scale (CPSS), a 10-item questionnaire, was used to measure perceived stress [28]. The Pittsburgh Sleep Quality Index (PSQI) [29] was used to assess sleep quality, with higher global scores indicating greater sleep dysfunction. Overall mood states and sub-scores (“fatigue-inertia,” “anger-hostility,” “vigor-activity,” “confusion-bewilderment,” “depression-dejection,” “tension-anxiety,” and “friendliness”) were evaluated using the Profile of Mood States (POMS) [30] questionnaire. Finally, a single-item, 10-point Likert scale was used to determine changes in subjective pain and discomfort experienced by participants (0: “No pain or discomfort” to 10: “Worst pain or discomfort”).

#### 2.4.5. Daily study diaries

Participants completed daily study diaries using the electronic data collection software, Medrio (San Francisco, California). Diaries were completed once per day following Visit 2 for the duration of the study period. Participants reported time of day the study product was consumed, if a high-fat food was consumed with the study product, supplement and medication consumption, any adverse events or changes in health, and if these health changes prompted any professional absences (i.e. work or school). Participant overall wellbeing was determined by a loss in professional productivity due to sick days recorded in the study diary.

### 2.5. Data analysis

Outcome efficacy measures were assessed using mixed effects models to evaluate changes from baseline (Visit 2) at Visits 3, 4, and 5, with the baseline value as a covariate, group and visit as fixed effects as well as the group x time interaction term, and participant ID as a random intercept. Analyses were conducted on the participants who had completed all assessments. An alpha level of 0.05 was used to determine statistical significance. The adjusted mean differences based on the results of the statistical model were used to calculate effect sizes as Cohen’s *d*. Due to the dearth of literature pertaining to the benefits of cannabidiol in healthy populations, trends are also reported to be transparent on statistical effects for future studies. Trends toward significance were reported at α ≤ 0.10. A secondary exploratory analysis was conducted to analyze results within each sex, based on prior research suggesting there may be sex differences in response to CBD supplementation. Effect sizes for the overall sample, as well as subdivided by males and females as a function of assessment time and group, were calculated from the unadjusted raw values also using Cohen’s *d* and presented in tabular form. Statistical analyses were performed using the statistical software R (Version 4.2.0).

## 3. Results

### 3.1. Participants

**Figure 2.**
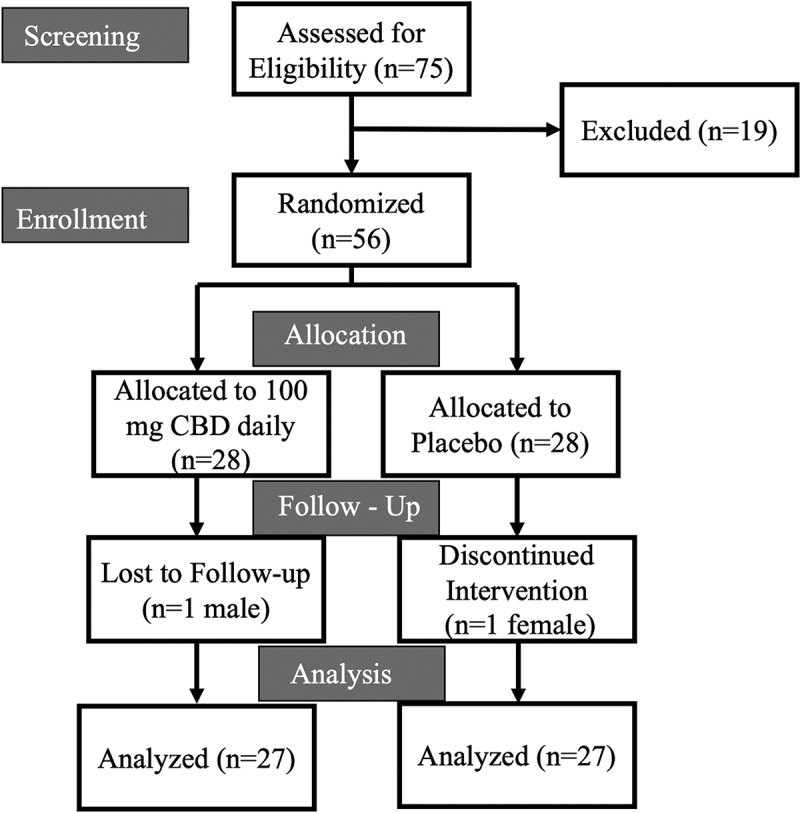
Consort diagram.

Seventy-five individuals were evaluated and screened, of which 56 were randomized (see Figure 2). Fifty-four participants completed the entire study protocol and were included in data analysis (Table 3). From Visit 2 to Visit 5, study product consumption compliance was 87.54%.

This diagram depicts the progress of participants through screening, enrollment, randomization into parallel groups, and follow-up.

**Table 3.** Baseline participant descriptive data.

	Total (*N* = 54)	Female (*n* = 27)	Male (*n* = 27)
Age (years)	25 ± 7	24 ± 8	24 ± 5
BMI (kg/m^2^)	24.82 ± 3.25	23.7 ± 3.4	26.1 ± 2.7
Non-Hispanic White (%)	85.2	70.4	81.5

Data are presented as mean ± SD.

### 3.2. Vital sign measures

No significant main effects of interaction effects were observed for heart rate, systolic, or diastolic blood pressure (*p* > 0.05).

### 3.3. Blood measures

No significant main effects or interaction effects were observed for serum ACE levels (*p* > 0.05) (see Table 4). Analyses revealed a main effect of Time on HCT levels (*p* = 0.019), which decreased from Visit 3 to Visit 4 (*p* = 0.021, *d* = 0.42), with no differences from Visit 3 to Visit 5 (*p* > 0.05, d = 0.33). No significant main effects or interactions were detected for the following immune system markers: WBC, RBC, HGB, MCV, mean platelet volume, neutrophils, lymphocytes, monocytes, eosinophils, and basophils (*p* > 0.05) (see Table 5). MCH and MCHC both had Group main effect trends (*p* = 0.076, *d*=-0.37 and *p* = 0.072, *d*=-0.38 respectively). It appeared that the group effect for MCH was primarily due to a higher value in the CBD group at Visit 4 (*p* = 0.078, *d* = 0.25), however, there were no significant effects or trends toward significance for post hoc tests of MCHC (*p* > 0.1). RDW had a Group main effect that trended toward significance (*p* = 0.096, *d*=-0.46), however none of the post hoc tests reached significance (*p* > 0.1). There was a trend for the Group main effect of platelet count (*p* = 0.087, *d* = 0.48), with post hoc tests showing the CBD group was higher at Visit 5 (*p* = 0.094, *d* = 0.65). Regarding inflammatory biomarkers, there were no Group or Time main effects or Group-by-Time interactions for TNF-α, IL-6, and IL-10 (*p* > 0.05) (see Table 4).

**Table 4.** Changes in blood biomarkers by group and sex.

Measure	Sex	CBD				Placebo					p-value	
		V2	V3	V4	V5	V2	V3	V4	V5	Time	Group	Interaction
**ACE** **(µKat/L)**	Overall	0.54 ± 0.27	0.56 ± 0.34(0.07)	0.58 ± 0.32(0.15)	0.58 ± 0.31(0.14)	0.60 ± 0.31	0.56 ± 0.22(0.13)	0.58 ± 0.27(0.06)	0.61 ± 0.24(0.03)	0.232	0.205	0.615
	Male	0.65 ± 0.26	0.68 ± 0.39(0.12)	0.68 ± 0.32(0.12)	0.68 ± 0.31(0.24)	0.65 ± 0.39	0.56 ± 0.26(0.23)	0.63 ± 0.34(0.05)	0.66 ± 0.29(0.03)	0.405	0.287	0.378
	Female	0.44 ± 0.26	0.44 ± 0.26(0.00)	0.46 ± 0.31(0.08)	0.48 ± 0.29(0.15)	0.56 ± 0.19	0.56 ± 0.17(0.00)	0.51 ± 0.17(0.26)	0.56 ± 0.19(0.00)	0.109	0.160	0.339
**TNF-a(pg/mL)**	Overall	14.1 ± 34.7	8.2 ± 9.9(0.17)	12.8 ± 25.5(0.04)	12.5 ± 23.4(0.05)	10.1 ± 13.1	7.1 ± 5.8(0.23)	7.6 ± 5.8(0.19)	7.6 ± 7.5(0.19)	0.301	0.277	0.461
	Male	9.4 ± 13.5	9.9 ± 13.3(0.04)	10.1 ± 12.5(0.05)	10.3 ± 13.2(0.07)	6.4 ± 2.7	6.0 ± 2.2(0.15)	6.6 ± 2.1(0.07)	6.4 ± 2.2(0.00)	0.423	0.025*	0.423
	Female	19.1 ± 48.8	6.4 ± 3.5(0.26)	15.8 ± 35.0(0.07)	15.0 ± 31.6(0.08)	15.3 ± 19.6	8.6 ± 8.8(0.34)	8.9 ± 8.8(0.33)	9.3 ± 11.6(0.31)	0.402	0.601	0.480
**IL-6(pg/mL)**	Overall	80 ± 161	58 ± 76(0.14)	87 ± 162(0.04)	82 ± 158(0.01)	55 ± 77	49 ± 59(0.08)	50 ± 62(0.06)	45 ± 61(0.13)	0.381	0.340	0.298
	Male	54 ± 71	57 ± 76(0.04)	59 ± 76(0.07)	57 ± 72(0.04)	36 ± 63	35 ± 60(0.01)	38 ± 65(0.03)	29 ± 60(0.11)	0.240	0.044*	0.525
	Female	113 ± 231	60 ± 81(0.23)	120 ± 230(0.03)	113 ± 225(0.00)	75 ± 90	65 ± 58(0.11)	62 ± 60(0.14)	63 ± 60(0.13)	0.354	0.684	0.302
**IL-10(pg/mL)**	Overall	14.3 ± 24.4	12.5 ± 18.3(0.07)	15.1 ± 24.3(0.03)	15.0 ± 23.8(0.03)	10.0 ± 11.5	9.2 ± 10.8(0.07)	9.2 ± 11.5(0.07)	9.3 ± 11.0(0.06)	0.476	0.240	0.490
	Male	13.8 ± 21.1	14.5 ± 21.6(0.03)	14.3 ± 20.8(0.02)	14.5 ± 20.2(0.03)	8.6 ± 14.1	7.9 ± 13.2(0.05)	8.3 ± 14.1(0.02)	8.1 ± 13.3(0.04)	0.978	0.013*	0.616
	Female	14.8 ± 29.0	10.0 ± 14.1(0.17)	16.0 ± 29.1(0.04)	15.6 ± 28.6(0.03)	11.3 ± 9.0	10.5 ± 8.2(0.09)	10.0 ± 9.0(0.14)	10.5 ± 8.7(0.09)	0.411	0.682	0.340

Data are presented as mean ± SD(*d*). All effect sizes (*d*) are compared to Visit 2 (V2).

*Denotes *p* < 0.05.

ACE = angiotensin converting enzyme, µKat/L = microkatals per liter, pg/mL = picogram per milliliter.

**Table 5.** Changes in immune system biomarkers by group and sex.

Measure	Sex	CBD				Placebo					p-value	
		V2	V3	V4	V5	V2	V3	V4	V5	Time	Group	Interaction
**WBC** **(10^3/μL)**	Overall	5.67 ± 1.50	5.80 ± 1.53(0.09)	5.85 ± 1.65(0.12)	5.65 ± 1.59(0.01)	5.13 ± 1.20	5.38 ± 1.00(0.21)	5.42 ± 1.37(0.24)	5.30 ± 1.13(0.14)	0.587	0.984	0.965
	Male	5.38 ± 1.43	5.42 ± 1.50(0.03)	5.34 ± 1.22(0.03)	4.97 ± 0.87(0.29)	4.76 ± 0.92	5.08 ± 0.93(0.35)	5.03 ± 1.27(0.29)	4.85 ± 0.91(0.10)	0.066#	0.384	0.757
	Female	5.95 ± 1.56	6.16 ± 1.53(0.13)	6.33 ± 1.88(0.24)	6.28 ± 1.86(0.21)	5.56 ± 1.39	5.74 ± 1.01(0.13)	5.89 ± 1.40(0.24)	5.85 ± 1.16(0.21)	0.852	0.654	0.999
**RBC** **(10^6/μL)**	Overall	4.76 ± 0.35	4.69 ± 0.41(0.20)	4.74 ± 0.42(0.06)	4.74 ± 0.41(0.06)	4.88 ± 0.32	4.83 ± 0.39(0.16)	4.90 ± 0.31(0.06)	4.87 ± 0.31(0.03)	0.197	0.467	0.876
	Male	4.99 ± 0.24	4.99 ± 0.3(0.00)	5.06 ± 0.27(0.29)	4.99 ± 0.32(0.00)	5.05 ± 0.31	5.03 ± 0.33(0.06)	5.02 ± 0.31(0.10)	5.00 ± 0.31(0.16)	0.499	0.512	0.471
	Female	4.56 ± 0.32	4.42 ± 0.30(0.44)	4.44 ± 0.30(0.38)	4.50 ± 0.34(0.19)	4.67 ± 0.20	4.59 ± 0.31(0.4)	4.76 ± 0.25(0.45)	4.70 ± 0.23(0.15)	0.141	0.079#	0.342
**HGB** **(g/dL)**	Overall	14.2 ± 1.1	14.3 ± 1.4(0.09)	14.3 ± 1.4(0.09)	14.2 ± 1.2(0.00)	14.1 ± 1.5	14.0 ± 1.5(0.07)	14.2 ± 1.4(0.07)	14.1 ± 1.5(0.00)	0.141	0.755	0.942
	Male	15.1 ± 0.5	15.2 ± 0.7(0.20)	15.3 ± 0.8(0.40)	15.1 ± 0.7(0.00)	15.2 ± 0.7	15.0 ± 0.7(0.29)	15.0 ± 0.7(0.29)	15.0 ± 0.8(0.29)	0.489	0.149	0.640
	Female	13.4 ± 0.7	13.1 ± 1.0(0.43)	13.3 ± 1.0(0.14)	13.3 ± 1.0(0.14)	12.9 ± 1.2	12.7 ± 1.2(0.17)	13.1 ± 1.5(0.17)	12.9 ± 1.4(0.00)	0.068#	0.674	0.656
**HCT** **(%)**	Overall	43.0 ± 2.9	42.5 ± 3.8(0.17)	43.2 ± 3.9(0.07)	42.9 ± 3.7(0.03)	42.8 ± 3.4	41.4 ± 5.0(0.41)	43.1 ± 3.3(0.09)	42.9 ± 3.4(0.03)	0.019*	0.780	0.432
	Male	45.5 ± 1.2	45.5 ± 2.2(0.00)	46.5 ± 2.3(0.83)	45.7 ± 2.3(0.17)	45.2 ± 2.2	43.2 ± 5.8(1.05)	45.1 ± 2.1(0.18)	45.0 ± 1.9(0.23)	0.169	0.103	0.584
	Female	40.7 ± 1.9	39.7 ± 2.6(0.53)	40.2 ± 2.1(0.26)	40.3 ± 2.6(0.21)	39.9 ± 2.2	39.4 ± 3.1(0.23)	40.7 ± 3.0(0.36)	40.4 ± 3.2(0.23)	0.063#	0.457	0.612
**MCV** **(fL)**	Overall	90.4 ± 4.4	90.6 ± 4.3(0.05)	91.3 ± 5.1(0.20)	90.7 ± 4.6(0.07)	87.6 ± 4.6	87.7 ± 4.5(0.02)	88 ± 4.9(0.09)	88.2 ± 5.4(0.13)	0.287	0.752	0.472
	Male	91.4 ± 4.1	91.4 ± 4.5(0.12)	91.9 ± 4.5(0.12)	91.7 ± 4.0(0.07)	89.6 ± 3.1	89.3 ± 3.0(0.10)	90.1 ± 3.5(0.16)	90.1 ± 3.7(0.16)	0.211	0.716	0.782
	Female	89.5 ± 4.6	89.8 ± 4.1(0.07)	90.7 ± 5.8(0.26)	89.8 ± 5.1(0.07)	85.3 ± 5.0	85.8 ± 5.4(0.16)	85.6 ± 5.4(0.04)	86.0 ± 6.5(0.04)	0.835	0.873	0.386
**MCH** **(pg)**	Overall	29.9 ± 2.0	30.1 ± 2.0(0.10)	30.2 ± 1.8(0.15)	30.0 ± 2.2(0.05)	29.0 ± 2.2	28.9 ± 2.1(0.05)	28.9 ± 2.3(0.05)	28.9 ± 2.4(0.05)	0.643	0.076#	0.674
	Male	30.3 ± 2.0	30.7 ± 2.0(0.20)	30.4 ± 1.8(0.05)	30.4 ± 1.9(0.05)	30.1 ± 1.0	29.9 ± 1.0(0.02)	30.0 ± 1.0(0.10)	30.0 ± 1.0(0.10)	0.476	0.160	0.325
	Female	29.6 ± 2.0	29.6 ± 1.9(0.00)	30.0 ± 2.0(0.20)	29.6 ± 2.4(0.00)	27.7 ± 2.6	27.6 ± 2.5(0.04)	27.6 ± 2.8(0.04)	27.5 ± 3.0(0.08)	0.510	0.336	0.632
**MCHC(g/dL)**	Overall	33.1 ± 0.9	33.4 ± 1.2(0.33)	33.0 ± 0.9(0.11)	33.0 ± 1.10(0.11)	33.1 ± 1.2	32.9 ± 1.2(0.17)	32.8 ± 1.3(0.25)	32.7 ± 1.4(0.33)	0.176	0.072#	0.745
	Male	33.2 ± 1.1	33.5 ± 0.9(0.27)	33.0 ± 0.6(0.18)	33.1 ± 1.0(0.09)	33.6 ± 0.8	33.5 ± 0.8(0.13)	33.3 ± 0.7(0.38)	33.3 ± 1.1(0.38)	0.092#	0.967	0.799
	Female	33.0 ± 0.8	33.3 ± 1.5(0.38)	33.1 ± 1.2(0.13)	32.9 ± 1.1(0.13)	32.4 ± 1.4	32.1 ± 1.1(0.21)	32.1 ± 1.6(0.21)	31.9 ± 1.5(0.36)	0.653	0.057#	0.876
**RDW** **(%)**	Overall	12.6 ± 0.7	12.5 ± 0.7(0.14)	12.4 ± 0.5(0.29)	12.4 ± 0.5(0.29)	12.8 ± 0.9	12.8 ± 0.9(0.00)	12.8 ± 0.9(0.00)	12.8 ± 0.9(0.00)	0.905	0.100	0.728
	Male	12.7 ± 0.7	12.4 ± 0.6(0.43)	12.4 ± 0.5(0.43)	12.3 ± 0.5(0.57)	12.5 ± 0.4	12.5 ± 0.4(0.00)	12.4 ± 0.4(0.25)	12.4 ± 0.4(0.25)	0.021*	0.147	0.899
	Female	12.6 ± 0.8	12.5 ± 0.8(0.13)	12.5 ± 0.5(0.13)	12.6 ± 0.5(0.00)	13.1 ± 1.3	13.1 ± 1.3(0.00)	13.2 ± 1.2(0.08)	13.3 + 1.1(0.15)	0.619	0.215	0.643
**Platelets (10^3/μL)**	Overall	248 ± 51	251 ± 52(0.06)	251 ± 64(0.06)	254 ± 58(0.12)	247 ± 69	241 ± 55(0.10)	245 ± 59(0.12)	235 ± 53(0.21)	0.718	0.087#	0.221
	Male	226 ± 38	233 ± 30(0.18)	222 ± 35(0.11)	236 ± 40(0.26)	218 ± 42	212 ± 32(0.14)	214 ± 39(0.10)	211 ± 34(0.17)	0.458	0.067#	0.149
	Female	270 ± 55	269 ± 64(0.02)	279 ± 74(0.16)	272 ± 69(0.04)	287 ± 80	279 ± 57(0.16)	285 ± 57(0.27)	266 ± 59(0.07)	0.161	0.407	0.454
**MPV** **(fL)**	Overall	10.9 ± 1.1	10.9 ± 1.0(0.00)	10.9 ± 1.0(0.00)	11.0 ± 1.0(0.09)	11.3 ± 1.1	11.2 ± 1.1(0.09)	11.3 ± 1.1(0.00)	11.3 ± 1.2(0.00)	0.317	0.742	0.998
	Male	11.0 ± 1.2	10.8 ± 1.1(0.17)	11.0 ± 1.1(0.00)	10.8 ± 1.1(0.17)	11.3 ± 1.3	11.3 ± 1.2(0.00)	11.3 ± 1.3(0.00)	11.4 ± 1.4(0.08)	0.752	0.198	0.305
	Female	10.9 ± 0.9	10.9 ± 0.9(0.00)	10.9 ± 0.8(0.00)	11.2 ± 0.9(0.33)	11.3 ± 0.9	11.1 ± 0.8(0.22)	11.2 ± 1.0(0.11)	11.2 ± 1.1(0.11)	0.316	0.533	0.678
**Neutrophils (10^3/μL)**	Overall	2.91 ± 1.1	2.89 ± 0.9(0.02)	3.07 ± 1.28(0.15)	2.72 ± 1.06(0.17)	2.69 ± 0.88	2.67 ± 0.72(0.02)	2.76 ± 0.79(0.08)	2.67 ± 0.8(0.02)	0.288	0.882	0.670
	Male	2.63 ± 0.99	2.75 ± 0.80(0.12)	2.66 ± 0.83(0.03)	2.49 ± 0.88(0.14)	2.52 ± 0.71	2.48 ± 0.64(0.06)	2.54 + 0.81(0.03)	2.46 ± 0.64(0.08)	0.358	0.707	0.529
	Female	3.18 ± 1.17	3.01 ± 1.00(0.15)	3.45 ± 1.51(0.23)	2.94 ± 1.19(0.21)	2.91 ± 1.05	2.9 ± 0.78(0.01)	3.01 ± 0.74(0.10)	2.92 ± 0.92(0.01)	0.365	0.853	0.644
**Lymphocytes (10^3/μL)**	Overall	2.00 ± 0.63	2.18 ± 0.76(0.29)	2.03 ± 0.57(0.05)	2.09 ± 0.93(0.14)	1.74 ± 0.5	1.95 ± 0.51(0.42)	1.91 ± 0.60(0.34)	1.85 ± 0.57(0.22)	0.448	0.856	0.711
	Male	1.91 ± 0.45	1.88 ± 0.56(0.24)	1.91 ± 0.43(0.00)	1.82 ± 0.44(0.20)	1.61 ± 0.36	1.81 ± 0.39(0.61)	1.75 ± 0.45(0.15)	1.67 ± 0.33(0.36)	0.299	0.442	0.729
	Female	2.07 ± 0.77	2.46 ± 0.83(0.51)	2.14 ± 0.66(0.09)	2.35 ± 1.18(0.36)	1.90 ± 0.61	2.10 ± 0.61(0.33)	2.10 ± 0.73(0.33)	2.06 ± 0.73(0.26)	0.607	0.607	0.584
**Monocytes (10^3/μL)**	Overall	0.51 ± 0.18	0.69 ± 1.00(0.18)	0.50 ± 0.13(0.06)	0.47 ± 0.11(0.22)	0.50 ± 0.12	0.49 ± 0.09(0.08)	0.48 ± 0.11(0.16)	0.48 ± 0.09(0.16)	0.354	0.458	0.432
	Male	0.54 ± 0.20	0.52 ± 0.17(0.10)	0.53 ± 0.13(0.05)	0.45 ± 0.09(0.45)	0.48 ± 0.14	0.49 ± 0.08(0.07)	0.48 ± 0.13(0.00)	0.47 ± 0.10(0.07)	0.047*	0.616	0.178
	Female	0.48 ± 0.16	0.85 ± 1.39(2.31)	0.47 ± 0.13(0.06)	0.48 ± 0.14(0.00)	0.51 ± 0.08	0.50 ± 0.11(0.13)	0.49 ± 0.1(0.25)	0.49 ± 0.07(0.25)	0.466	0.429	0.522
**Eosinophils (10^3/μL)**	Overall	0.20 ± 0.16	0.20 ± 0.14(0.00)	0.20 ± 0.12(0.00)	0.33 ± 0.72(0.81)	0.18 ± 0.14	0.21 ± 0.22(0.21)	0.21 ± 0.19(0.21)	0.22 ± 0.21(0.29)	0.443	0.809	0.541
	Male	0.24 ± 0.2	0.23 ± 0.19(0.05)	0.19 ± 0.13(0.25)	0.17 ± 0.10(0.35)	0.19 ± 0.16	0.24 ± 0.29(0.31)	0.20 ± 0.20(0.06)	0.23 ± 0.22(0.25)	0.219	0.141	0.494
	Female	0.15 ± 0.10	0.17 ± 0.07(0.20)	0.20 ± 0.12(0.50)	0.47 ± 1.00(3.2)	0.17 ± 0.13	0.18 ± 0.09(0.08)	0.23 ± 0.19(0.46)	0.21 ± 0.20(0.31)	0.399	0.397	0.439
**Basophils (10^3/μL)**	Overall	0.07 ± 0.14	0.06 ± 0.10(0.07)	0.04 ± 0.02(0.21)	0.05 ± 0.08(0.14)	0.04 ± 0.02	0.04 ± 0.03(0.00)	0.05 ± 0.03(0.5)	0.04 ± 0.03(0.00)	0.588	0.535	0.400
	Male	0.03 ± 0.02	0.06 ± 0.10(1.50)	0.03 ± 0.01(0.00)	0.07 ± 0.10(2.0)	0.04 ± 0.02	0.04 ± 0.03(0.00)	0.04 ± 0.03(0.00)	0.04 ± 0.03(0.00)	0.567	0.209	0.453
	Female	0.11 ± 0.02	0.07 ± 0.1(2.00)	0.04 ± 0.02(3.50)	0.03 ± 0.02(4.00)	0.04 ± 0.02	0.04 ± 0.02(0.00)	0.05 ± 0.02(0.05)	0.04 ± 0.03(0.00)	0.393	0.497	0.750

Data are presented as mean ± SD(*d*). All effect sizes (*d*) are compared to Visit 2 (V2).

*Denotes *p* < 0.05.

#Denotes *p* < 0.1.

WBC = white blood cells, RBC = red blood cells, HGB = hemoglobin, HCT = hematocrit, MCV = mean corpuscular volume, MCH = mean corpuscular hemoglobin, MCHC = mean corpuscular hemoglobin concentration, RDW = RBC distribution width, MPV = mean platelet volume, µL = microliter, pg = picogram, g/dL = grams per deciliter, fL= femtoliter.

### 3.4. Questionnaires

Analyses revealed no main effects or Group-by-Time interactions for CPSS, PSQI, overall mood disturbance, or the POMS subscales (*p* > 0.05), except “vigor-activity” (see Tables 6 & 7). A Time main effect was found for the sub-score for “vigor” (*p* = 0.007), which decreased from Visit 3 to Visit 4 (*p* = 0.025, *d*=-0.38) and from Visit 3 to Visit 5 (*p* = 0.014, *d*=-0.41). A trend toward significance was found for a Group main effect of the body discomfort scale (*p* = 0.089, *d*=-0.48), with the CBD group reporting lower discomfort at Visits 3 (*p* = 0.072, *d*=-0.30) and 5 (*p* = 0.023, *d* = 0.62). No significant differences were found between groups for overall well-being (*p* > 0.05) (Table 8).

**Table 6.** Changes in perceived stress, sleep quality, and body discomfort by group and sex.

Measure	Sex	CBD				Placebo					p-value	
		V2	V3	V4	V5	V2	V3	V4	V5	Time	Group	Interaction
**CPSS**	Overall	14.5 ± 7.6	12.2 ± 7.7(0.30)	13.1 ± 6.6(0.18)	13.4 ± 6.8(0.14)	11.9 ± 5.9	12.7 ± 6.3(0.14)	12.7 ± 6.7(0.14)	12.2 ± 7.5(0.05)	0.792	0.204	0.456
	Male	10.8 ± 5.7	9.9 ± 6.3(0.15)	10.5 ± 6.1(0.05)	10.7 ± 5.0(0.01)	12.0 ± 5.7	11.9 ± 7.0(0.02)	11.6 ± 6.5(0.07)	10.3 ± 5.7(0.30)	0.793	0.954	0.423
	Female	18.0 ± 7.7	14.3 ± 8.5(0.48)	15.4 ± 6.4(0.33)	15.9 ± 7.5(0.27)	11.9 ± 6.4	13.5 ± 5.7(0.25)	13.8 ± 6.9(0.30)	14.2 ± 8.8(0.36)	0.570	0.047*	0.874
**PSQI**	Overall	5.48 ± 2.99	5.22 ± 2.94(0.09)	5.3 ± 3.12(0.06)	5.07 ± 2.6(0.14)	4.89 ± 2.49	4.26 ± 2.38(0.25)	4.48 ± 2.42(0.16)	4.37 ± 2.79(0.21)	0.815	0.337	0.815
	Male	4.31 ± 2.10	3.62 ± 2.33(0.33)	4.00 ± 2.20(0.15)	3.85 ± 2.08(0.22)	4.57 ± 2.85	4.29 ± 2.52(0.10)	3.86 ± 2.25(0.25)	4.07 ± 2.30(0.18)	0.996	0.864	0.533
	Female	6.57 ± 3.34	6.71 ± 2.70(0.04)	6.50 ± 3.44(0.02)	6.21 ± 2.58(0.11)	5.23 ± 2.09	4.23 ± 2.31(0.48)	5.15 ± 2.51(0.04)	4.69 ± 3.30(0.26)	0.642	0.205	0.395
**Body Discomfort**	Overall	1.30 ± 1.10	1.26 ± 1.23(0.04)	1.22 ± 1.15(0.07)	1.04 ± 1.13(0.24)	0.81 ± 1.11	1.30 ± 1.38(0.44)	1.11 ± 1.34(0.27)	1.44 ± 1.72(0.57)	0.812	0.090#	0.318
	Male	1.38 ± 1.26	1.23 ± 1.48(0.12)	1.38 ± 1.12(0.00)	1.15 ± 1.28(0.18)	0.79 ± 1.12	1.21 ± 1.42(0.38)	1.36 ± 1.65(0.51)	1.57 ± 2.03(0.70)	0.862	0.108	0.709
	Female	1.21 ± 0.97	1.29 ± 0.99(0.08)	1.07 ± 1.21(0.14)	0.93 ± 1.00(0.29)	0.85 ± 1.14	1.38 ± 1.39(0.46)	0.85 ± 0.90(0.00)	1.31 ± 1.38(0.40)	0.118	0.449	0.249

Data are presented as mean ± SD(*d*). All effect sizes (*d*) are compared back to Visit 2 (V2).

*Denotes *p* < 0.05.

#Denotes *p* < 0.1.

CPSS= Cohens perceived stress scale, PSQI = Pittsburgh sleep quality index.

**Table 7.** Changes in profile of mood states total mood disturbance and subscales by group and sex.

Measure	Sex	CBD				Placebo					p-value	
		V2	V3	V4	V5	V2	V3	V4	V5	Time	Group	Interaction
**Total Mood Disturbance**	Overall	19.3 ± 26.7	18.3 ± 28.4(0.04)	23.5 ± 29.0(0.16)	20.4 ± 29.5(0.04)	14.3 ± 23.4	13.3 ± 25.8(0.04)	16.1 ± 25.8(0.08)	17.6 ± 34.3(0.14)	0.298	0.842	0.694
	Male	9.9 ± 25.3	11.0 ± 25.5(0.04)	18.1 ± 28.0(0.32)	17.5 ± 31.1(0.30)	15.0 ± 28.4	12.1 ± 27.7(0.10)	13.0 ± 28.9(0.07)	14.2 ± 30.1(0.03)	0.446	0.396	0.732
	Female	28.1 ± 25.8	25.1 ± 30.3(0.12)	29.4 ± 29.7(0.05)	23.1 ± 28.9(0.19)	13.5 ± 17.7	14.6 ± 24.6(0.06)	19.4 ± 22.7(0.33)	21.2 ± 39.2(0.44)	0.545	0.319	0.509
**Anger Hostility**	Overall	5.00 ± 4.51	5.11 ± 5.26(0.02)	5.89 ± 5.69(0.20)	5.15 ± 5.77(0.03)	3.63 ± 3.85	3.19 ± 3.37(0.11)	2.96 ± 4.64(0.17)	3.26 ± 4.90(0.10)	0.853	0.178	0.532
	Male	5.62 ± 4.94	6.31 ± 5.60(0.23)	8.15 ± 5.11(0.43)	6.69 ± 5.79(0.19)	4.29 ± 4.53	3.93 ± 3.60(0.12)	3.71 ± 5.33(0.12)	3.64 ± 5.08(0.15)	0.532	0.081#	0.436
	Female	4.43 ± 4.18	4.00 ± 4.85(0.10)	3.79 ± 5.54(0.15)	3.71 ± 5.57(0.17)	2.92 ± 2.99	2.38 ± 3.04(0.18)	2.15 ± 3.80(0.26)	2.85 ± 4.86(0.02)	0.893	0.925	0.811
**Confusion Bewilderment**	Overall	9.30 ± 6.28	9.04 ± 6.04(0.04)	10.19 ± 6.59(0.14)	9.89 ± 5.81(0.09)	8.26 ± 4.98	8.22 ± 5.67(0.01)	9.15 ± 5.44(0.18)	9.00 ± 6.88(0.15)	0.221	0.839	0.984
	Male	7.38 ± 4.68	7.00 ± 4.76(0.08)	8.23 ± 4.97(0.18)	8.54 ± 4.97(0.25)	8.07 ± 6.11	7.71 ± 5.99(0.06)	8.64 ± 6.34(0.09)	9.07 ± 6.41(0.16)	0.210	0.952	0.984
	Female	11.07 ± 7.18	10.93 ± 6.63(0.02)	12.00 ± 7.53(0.13)	11.14 ± 5.67(0.01)	8.46 ± 3.64	8.77 ± 5.48(0.09)	9.69 ± 4.46(0.34)	8.92 ± 7.61(0.13)	0.539	0.736	0.999
**Depression Dejection**	Overall	4.67 ± 4.92	5.67 ± 7.50(0.20)	6.15 ± 6.57(0.30)	4.89 ± 7.00(0.04)	4.89 ± 6.22	5.07 ± 6.88(0.03)	4.41 ± 4.65(0.08)	5.81 ± 7.04(0.15)	0.991	0.572	0.181
	Male	3.62 ± 4.23	5.08 ± 5.57(0.35)	6.77 ± 7.25(0.74)	6.00 ± 7.77(0.56)	5.79 ± 8.04	6.43 ± 7.95(0.08)	5.00 ± 5.90(0.10)	6.36 ± 6.98(0.07)	0.909	0.396	0.286
	Female	5.64 ± 5.46	6.21 ± 9.12(0.10)	5.57 ± 6.10(0.01)	3.86 ± 6.31(0.32)	3.92 ± 3.45	3.62 ± 5.45(0.09)	3.77 ± 2.89(0.04)	5.23 ± 7.35(0.38)	0.936	0.645	0.140
**Fatigue Inertia**	Overall	8.48 ± 6.06	7.85 ± 5.57(0.10)	8.41 + 5.54(0.01)	7.67 ± 5.55(0.13)	6.63 ± 3.41	6.70 ± 3.36(0.02)	6.93 ± 4.36(0.09)	7.15 ± 5.45(0.15)	0.742	0.791	0.635
	Male	6.62 ± 5.80	6.15 ± 5.27(0.08)	6.08 ± 4.31(0.09)	6.15 ± 4.88(0.08)	7.00 ± 3.49	6.50 ± 3.30(0.14)	6.57 ± 4.88(0.12)	6.36 ± 4.5(0.18)	0.993	0.911	0.977
	Female	10.21 ± 5.98	9.43 ± 5.54(0.13)	10.57 ± 5.81(0.06)	9.07 ± 5.93(0.19)	6.23 ± 3.42	6.92 ± 3.55(0.20)	7.31 ± 3.88(0.32)	8.00 ± 6.39(0.52)	0.629	0.429	0.377
**Tension Anxiety**	Overall	10.93 ± 7.69	10.11 ± 6.89(0.11)	11.15 ± 7.34(0.03)	10.15 ± 7.90(0.10)	9.89 ± 6.46	9.30 ± 6.31(0.09)	9.85 ± 6.50(0.01)	10.30 ± 8.31(0.06)	0.520	0.881	0.582
	Male	6.77 ± 5.28	7.31 ± 5.22(0.10)	8.23 ± 4.90(0.28)	8.38 ± 5.99(0.30)	9.43 ± 7.06	8.14 ± 6.21(0.18)	8.07 ± 6.11(0.19)	9.00 ± 6.97(0.06)	0.551	0.359	0.840
	Female	14.79 ± 7.69	12.71 ± 7.38(0.27)	13.86 ± 8.31(0.12)	11.79 ± 9.26(0.39)	10.38 ± 5.99	10.54 ± 6.41(0.03)	11.77 ± 6.58(0.23)	11.69 ± 9.63(0.22)	0.509	0.077#	0.579
**Vigor Activity**	Overall	19.1 ± 5.2	19.5 ± 5.5(0.08)	18.3 ± 5.6(0.15)	17.3 ± 5.3(0.35)	19.0 ± 5.7	19.2 ± 6.4(0.04)	17.2 ± 7.3(0.32)	17.9 ± 7.1(0.19)	0.008*	0.789	0.369
	Male	20.2 ± 5.7	20.9 ± 7.0(0.12)	20.4 ± 5.9(0.04)	18.3 ± 6.4(0.33)	19.6 ± 6.1	7.7 ± 6.0(1.95)	8.6 ± 6.3(1.8)	9.1 ± 6.4(1.72)	0.178	0.620	0.114
	Female	18.1 ± 5.0	17.6 ± 5.9(0.10)	16.4 ± 4.7(0.37)	16.4 ± 4.0(0.37)	16.5 ± 5.0	17.6 ± 5.9(0.22)	15.3 ± 7.1(0.24)	15.5 ± 7.2(0.20)	0.038*	0.355	0.964
**Friendliness**	Overall	16.9 ± 3.6	16.0 ± 3.4(0.25)	16.2 + 4.3(0.19)	15.3 ± 4.0(0.44)	17.9 ± 3.5	17.9 ± 3.7(0.00)	16.9 ± 4.1(0.29)	17.1 ± 3.9(0.23)	0.179	0.204	0.291
	Male	17.3 ± 4.3	16.1 ± 4.7(0.28)	16.7 ± 5.1(0.14)	15.1 ± 5.2(0.51)	17.6 ± 3.3	17.6 ± 3.9(0.00)	16.6 ± 3.4(0.30)	17.4 ± 3.5(0.06)	0.559	0.250	0.156
	Female	16.6 ± 3.0	15.9 ± 1.9(0.23)	15.6 ± 3.5(0.33)	15.6 ± 2.6(0.33)	18.1 ± 3.8	18.2 ± 3.6(0.03)	17.2 ± 4.9(0.24)	16.9 ± 4.5(0.32)	0.218	0.526	0.218

Data are presented as mean ± SD(*d*). All effect sizes (*d*) are compared back to Visit 2 (V2).

*Denotes *p* < 0.05.

#Denotes *p* < 0.1.

**Table 8.** Changes in missed productivity by group and sex.

Measure	Sex	CBD			Placebo			p-value		
		V2 to V3	V3 to V4	V4 to V5	V2 to V3	V3 to V4	V4 to V5	Time	Group	Interaction
**Missed Productivity**	Overall	0 ± 0	0 ± 1	0 ± 0	0 ± 0	0 ± 1	0 ± 1	0.326	0.756	0.256
	Male	0 ± 0	0 ± 0	0 ± 0	0 ± 0	0 ± 0	0 ± 1	0.726	0.999	0.726
	Female	0 ± 1	0 ± 1	0 ± 1	0 ± 1	0 ± 1	1 ± 2	0.905	0.709	0.317

Data are presented as mean ± SD.

### 3.5. Pain index

There was a Group main effect for FPI (*p* = 0.028, *d*=-0.64) when adjusting for baseline values, indicating the PL group had a greater pain index over the intervention compared to the CBD group (Table 9).

**Table 9.** Changes in subscales of foundational pain index by group and sex.

Measure	Sex	CBD				Placebo					p-value	
		V2	V3	V4	V5	V2	V3	V4	V5	Time	Group	Interaction
**Foundational Pain Index**	Overall	11.9 ± 14.4	8.8 ± 11.7(0.22)	13.2 ± 10.4(0.09)	8.8 ± 10.9(0.22)	9.0 ± 14.2	14.4 ± 16.3(0.38)	15.6 ± 14.7(0.46)	12.9 ± 11.5(0.27)	0.116	0.028*	0.669
	Male	11.5 ± 16.8	8.9 ± 12.2(0.15)	13.7 ± 10.3(0.13)	10.7 ± 10.9(0.05)	4.7 ± 9.1	12.9 ± 17.2(0.90)	9.0 ± 13.3(0.47)	11.2 ± 12.7(0.71)	0.971	0.242	0.142
	Female	12.3 ± 12.3	8.7 ± 11.7(0.29)	12.7 ± 10.9(0.03)	6.9 ± 11.0(0.44)	14.0 ± 17.6	16.2 ± 15.7(0.13)	23.4 ± 12.7(0.53)	14.8 ± 10.2(0.05)	0.037*	0.023*	0.833

Data are presented as mean ± SD(*d*). All effect sizes (*d*) are compared back to Visit 2 (V2).

*Denotes *p* < 0.05.

### 3.6. Male-specific results

Twenty-seven males were included in this analysis (age = 24 ± 5y; BMI = 26.1 ± 2.7 kg/m^2^). A Time main effect was found for RDW (*p* = 0.021) from Visit 3 to Visit 5 (*p* = 0.015, *d*=-0.47). A Group main effect was observed when controlling for baseline values of TNF-α (*p* = 0.025, *d* = 0.94), IL-10 (*p* = 0.013, *d* = 1.22), and IL-6 (*p* = 0.043, *d* = 0.93), with overall lower values for PL. WBC indicated a Time main effect trend (*p* = 0.066), with no significant post hoc tests (*p* > 0.1). MCHC had a Time main effect trend (*p* = 0.092) with no significant post hoc tests (*p* > 0.1). An Interaction trend was found for monocyte count (*p* = 0.065) with no significant post hoc tests (*p* > 0.1). No significant interaction or main effects were found in any other blood biomarkers for males (*p* > 0.05) and there were no differences in any questionnaires or FPI (*p* > 0.05). There was a Group main effect trend toward significance for the “anger-hostility” subscale (*p* = 0.081, *d* = 0.71), with the PL group having lower levels of “anger-hostility” at Visit 4.

### 3.7. Female-specific results

This analysis included 27 females (age = 24 ± 8y; BMI = 23.7 ± 3.4 kg/m^2^). There was a trend toward a Group main effect for RBC (*p* = 0.079, *d*=-0.77), with post hoc testing showing CBD RBC was higher at Visit 4 (*p* = 0.080, *d*=-0.81). HGB and HCT had Time main effect trends toward significance (*p* = 0.068 and *p* = 0.63, respectively), but no significance was found in post hoc testing (*p* > 0.1). No differences were found in the female subgroup analyses for any other blood biomarkers (*p* > 0.05). The female subgroup showed no differences in their overall well-being (*p* > 0.05). A significant Group main effect was found for perceived stress (*p* = 0.047, *d*=-0.84) at Visits 3 (*p* = 0.007, *d*=-0.79) and 4 (*p* = 0.024, *d=−0.61*), indicating those in the CBD group had higher stress levels. A Time main effect was found in females for the mood subscale of “vigor-activity” (*p* = 0.038), with a decrease from Visit 3 to 4 (*p* = 0.058, *d*=-0.50) and Visit 3 to 5 (*p* = 0.077, *d*=-0.47). The main effects of both Group (*p* = 0.023, *d*=-1.01) and Time (*p* = 0.037) were shown in the FPI, indicating the PL group had a greater pain index than the CBD group and there was a decrease in FPI from Visit 4 to 5 (*p* = 0.038, *d*=-0.64). “Tension-anxiety” had a Group main effect trend (*p* = 0.077, *d*=-0.73), with lower scores in the PL group. No differences were observed in sleep quality, body discomfort, overall mood disturbance, or the rest of the subscales (*p* > 0.05).

## 4. Discussion

To our knowledge, this is the first study to rigorously evaluate a wide range of health effects of a hemp-derived CBD product in healthy adults over 12 weeks. Study results indicated CBD was safe and well-tolerated by all participants in this study. While there were no Group by Time interaction effects in the overall sample pool, there was a decrease in HCT and the POMS subscale for “vigor-activity.” Pain index, as indicated by FPI scores, was increased in the PL over the intervention compared to the CBD group, and a similar trend was found for body discomfort, with the CBD group reporting lower discomfort at Visit 3 and Visit 5. There were trends observed toward higher RDW in the PL group as well as higher MCH and platelet counts in the CBD group. Previous work on CBD has demonstrated mixed results, and much of these data come from diseased populations, case studies, open-label trials, or acute doses, leading to difficulties in application to chronic use in healthy individuals.

Additionally, while exploratory analyses based on sex revealed some interesting potential differences, future research should aim to elucidate possible sex differences. In males, there were lower inflammatory markers in the PL group. While there were no statistically significant differences in baseline measures between groups, at an absolute level, the male CBD group entered the study with higher cytokines than the PL group, potentially indicating CBD made no difference in cytokine levels throughout the study. Additionally, “anger-hostility” was lower for males in the PL group and there was a trend for higher WBC for males in the PL group. Females in the CBD group reported higher perceived stress, and higher “tension-anxiety” than the PL group. There was a greater decrease over the course of the intervention within the female CBD group for pain index measured by way of the FPI.

While CBD is understood to affect ACE2 activity in in vitro lung tissue directly [8], these effects may not be clinically significant as we did not find any differences in ACE activity. Furthermore, it is possible that the 100 mg CBD dosage or the 12-week study duration was insufficient to produce significant effects on ACE values. Additionally, it is possible that CBD does not produce a meaningful effect on ACE activity in healthy adults. The absence of differences between groups for missed productivity days, with low values overall, limits our ability to conclude the effectiveness of CBD in alleviating sickness.

The present study found the PL group experienced an increase in pain index throughout the intervention. At the same time, the CBD group’s pain index decreased as indicated by the FPI. Those in the CBD group also reported a trend toward less body discomfort than those in the PL group. Of note, both body discomfort and pain index were considered low in both groups throughout the intervention, which is to be expected in this population. Few studies have explored the effects of CBD alone on pain biomarkers or self-reported pain and those that have generally report mixed results [31–33]. A meta-analysis demonstrated a small effect for the use of cannabinoids when treating chronic pain [34], yet it is hard to extrapolate these results for CBD alone as the studies usually administered products containing a mixture of THC and low-dosage CBD (i.e. 15.7 mg or 2.5 mg of CBD) in clinical populations [35–37]. The effect of CBD supplementation on pain index demonstrated in the present study may be due to the fact that we administered a higher dosage as compared to prior studies. This area of research is in its early stages, and more work needs to be conducted to elucidate the effect of CBD alone on pain index.

The results indicating lower plasma concentrations of TNF- α, IL-10, and IL-6 in the male PL sub-group are presumably due to the CBD group entering the study with elevated absolute cytokine values compared to the PL group, the latter of which had no real changes throughout the study duration. The lack of true change seen in the present study may be rationalized by prior work demonstrating the anti-inflammatory effects of CBD utilizing much higher doses (>10 mg/kg). Additionally, the sample employed in this study was screened to ensure normal baseline levels of inflammation, leaving little room for improvement. Studies investigating specific clinical populations where inflammatory biomarkers are abnormal may provide evidence to support the anti-inflammatory role of CBD. Future studies should investigate acute uses of CBD in otherwise healthy populations when inflammatory markers are elevated, such as with intense exercise, acute stressors, or environmental exposure.

While self-reported surveys report 65% of respondents use CBD to relieve stress [38], the present study found no differences in perceived stress in the overall sample. However, when specifically investigating sex-based responses, there was a decrease in perceived stress levels in the female CBD group, yet the CBD group had higher reported stress throughout the study than the PL group. The CBD group also reported higher POMS subscale “tension-anxiety” scores than the PL group. Typically, human studies investigating the anxiolytic properties of CBD have utilized acute experimental manipulation of stress-inducing situations [2]. For example, when 300 mg of CBD was administered prior to a Simulated Public Speaking test in healthy individuals, a significant decrease in anxiety rating was found compared to placebo groups [39]. A possible explanation for the current findings is a U-shaped dose-response curve for CBD, studies found that a middle/moderate dose of CBD (300 mg) reduced anxiety, while low doses of CBD (100, 150 mg) and high doses (600, 900 mg) did not improve anxiety following public speaking [39,40]. The present study utilized 100 mg per day of CBD, which would fall into the low-dosing category. Additionally, this was a healthy population that consistently scored as “low stress” on the CPSS, without experimental manipulation to induce stress. Although exploratory, an interesting finding was the decrease in perceived stress in the CPSS observed only in the female CBD group. Data from preclinical and clinical studies indicates that females may experience an increased plasma CBD concentration compared to healthy males [41]. It has been postulated that this may be linked to hormonal differences between males and females as estradiol has been reported to modulate CB1 receptor density and affinity [42,43]. The current study’s investigation of sex-specific outcomes was an exploratory secondary outcome measure; therefore, future research should focus on identifying these potential sex-specific treatment effects as a main outcome measure.

There were no changes in total mood disturbance, with similar results reported among healthy participants consuming acute doses of 300, 600, and 900 mg of CBD [44] and in healthy individuals using 300 mg of CBD before exercise [45]. However, an outpatient adult population with anxiety had a 63.67% decrease in total mood disturbance with CBD [15]. Examination of the POMS subscore, “vigor-activity,” for the overall sample revealed significant reductions over time. This may have been attributed to the timing of the study in relation to the academic year. In males of the PL group, though there was a decrease in “anger-hostility,” the scores in both groups were low out of the total possible score. Additionally, both the mean scores and changes within each subscale score were similar to values found in healthy young adults [46].

No changes in sleep quality scores were found over three months in the current study. This is consistent with recent literature [40,47], which found no changes in PSQI with CBD administration. Carlini et al., initially reported 160 mg of CBD improved sleep quality measured by duration and interruptions in 15 healthy volunteers but did not find any differences in time to fall asleep [48]. Much of the literature on the hypnotic effects of CBD has been conducted as case studies in individuals with comorbidities which makes it difficult to extrapolate these findings to healthy individuals [49,50]. While increased sleep is one of the main reasons individuals use CBD, it may have little to no effect on otherwise healthy individuals. Future studies may want to investigate the use of CBD on sleep with more objective measures, such as using polysomnography, and in populations with difficulty sleeping.

### 4.1. Strengths and limitations

This study had several notable strengths. The longitudinal, counterbalanced, placebo-controlled, double-blinded design was a major strength of the research. Compliance was measured through daily reporting and objective measurements of returned products. This study is readily generalizable to the larger population as it was a free-living study investigating healthy adults. Prior CBD literature is mostly in clinical populations, leaving gaps in the understanding of the effects of CBD in healthy adults. As participants were evaluated at monthly intervals, female participants were likely tested at similar phases in the menstrual cycle to reduce the chances of changes among females occurring due to cycling hormone levels. Prior work in female athletes has conducted a similar approach of testing in 4-week spans to “control” for hormonal changes [51,52]. Lastly, objective health biomarkers were used to further ensure the safety of 100 mg daily doses of CBD over 12 weeks.

While the current study has many strengths, it is not without its limitations. The CBD dose used in this study was lower than some previously reported efficacious doses to ensure participants consumed quantities of the product that were below previously established upper safety limits and to remain consistent with the dosing guidance of the product being investigated. Participants took the product home and consumed it without observation, which introduced the potential for noncompliance. However, this risk was deemed acceptable compared to the risk of attrition that may have resulted from requiring participants to report to the study site twice daily. A relative dose based on body weight may have led to a greater incidence of statistically significant results for the outcome measures, as an absolute dose was used for all participants in this study. However, this absolute dose approach is more consistent with typical recommendations or guidance for CBD use. Additionally, pharmacokinetic data would be valuable to aid in understanding sex-specific responses. It is also possible that some of the measures used were not sensitive enough to detect chronic changes. Lastly, including cortisol as an objective stress biomarker could provide additional insight of participant stress levels.

## 5. Conclusion

In the present study, brand-specific, hemp-derived CBD was efficacious in reducing pain index scores in both men and women. Interestingly, significant sex-specific effects were found: males in the PL group had lower levels of inflammatory markers and “anger-hostility” scores, and females in the CBD group experienced lower pain index scores and lower levels of “tension-anxiety,” and had higher levels of “vigor-activity.” No serious adverse events were reported suggesting the product and dose was safe and well-tolerated in healthy adults. The lower frequency of significant outcome measures and lack of any sign of negative health outcome indicates higher CBD doses should be explored for these outcome measures in healthy populations. It is possible higher doses are needed to exhibit effects in healthy individuals or there may be a ceiling effect of the therapeutic potential of CBD in these populations. Exploratory outcomes based on sex are intriguing, however more work on the mechanisms of these effects is needed.
